# Supplementary material for: Hyperactivated PTP1B phosphatase in parvalbumin neurons alters anterior cingulate inhibitory circuits and induces autism-like behaviors
Source: Nat Commun. 2020 Feb 24;11:1017. doi: 10.1038/s41467-020-14813-z (PMC7039907; doi:10.1038/s41467-020-14813-z)
Supplement: Supplementary file 1 — Supplementary Information [file 41467_2020_14813_MOESM1_ESM.pdf]

Unleashed tyrosine phosphatase PTP1B activity in parvalbumin neurons alters homeostasis of anterior cingulate inhibitory circuits and induces autism-like behaviors in mice

Li Zhang et al.

Supplementary information

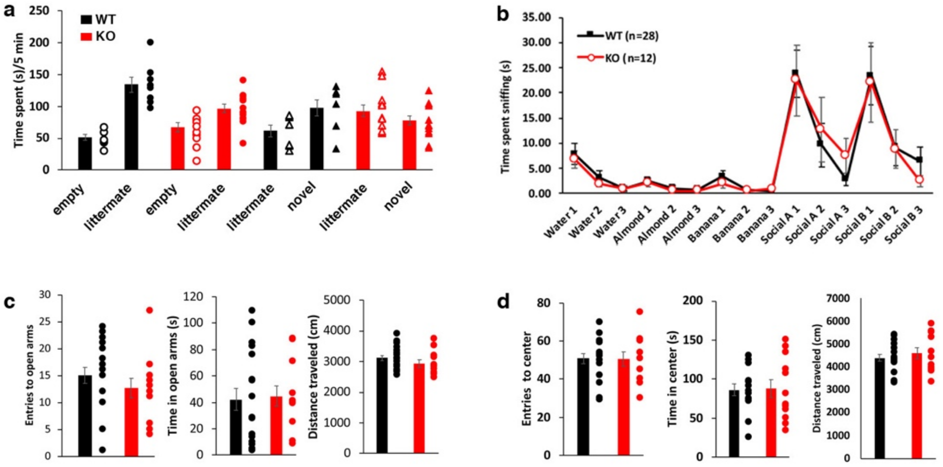

Supplementary Figure 1 Behavior tests of PV-*Lmo4*KO mice. (**a**) Time spent in social interaction and novelty tests, used for discrimination index in Figure 1. n=8 WT, 14 KO. (**b**) Olfactory response measured as time spent sniffing non-social and social odors. n=28 WT, 12 KO. (**c**) Elevated plus maze test. (**d**) Open field test. n=18 WT, 12 KO (c, d). All data are presented as means ± SEM.

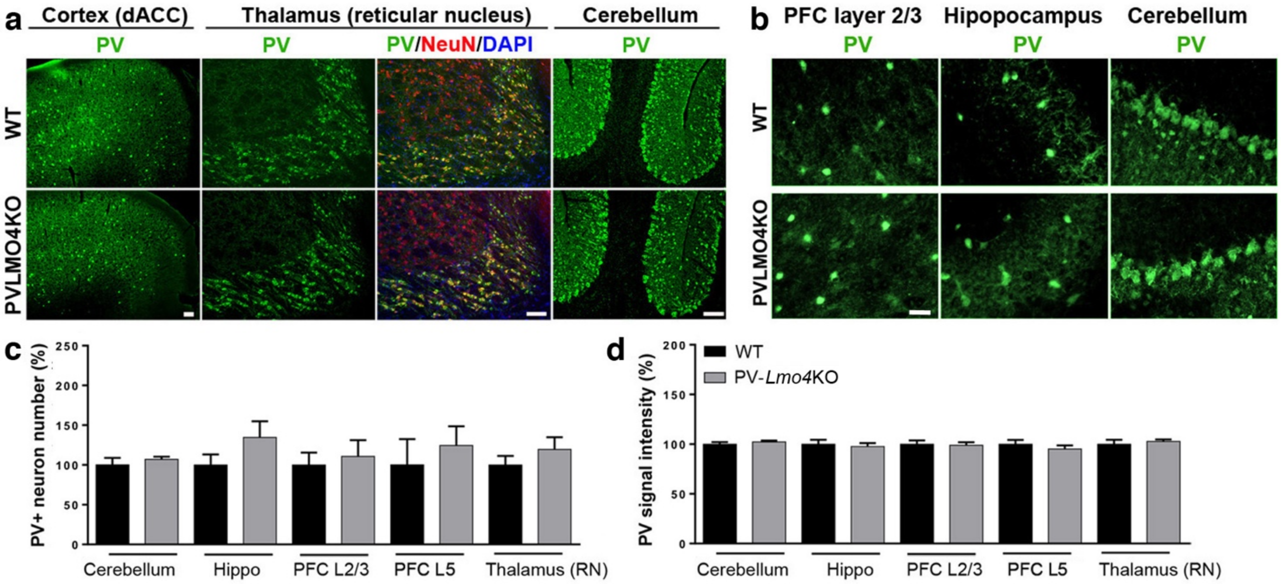

Supplementary Figure 2 No loss of PV neurons or PV expression in PV-*Lmo4*KO mice. PV-*Lmo4*KO mice show similar PV+ immunostaining (**a**, **b**) in numbers (**c**) and density (**d**) as littermate control WT mice. Scale bar, 100 μm (**a**) and 35 μm (**b**). n=4 mice per genotype. All data are presented as means ± SEM.

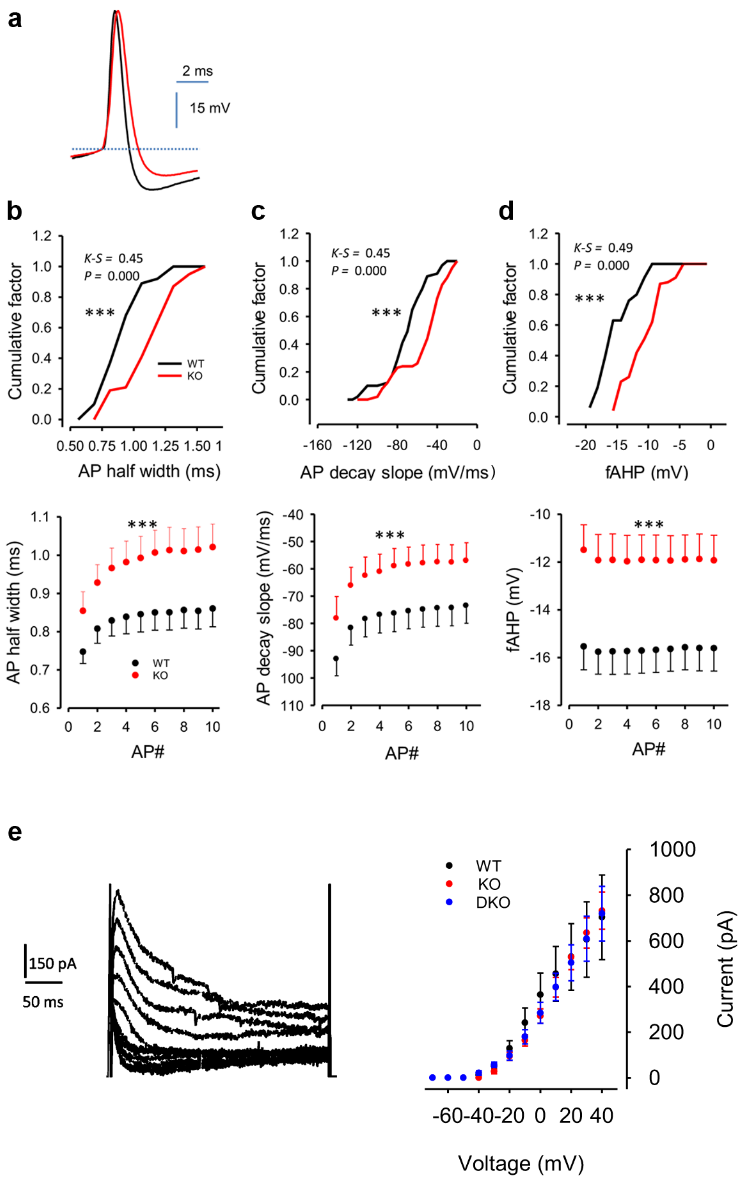

Supplementary Figure 3 PV interneuron properties are altered in PV-*Lmo4*KO mice. (**a**) Representative action potential (AP) traces of WT (black) and KO (red). Distribution of the parameters (AP half width (**b**), AP decay slope (**c**), fAHP (**d**)) from all AP (top panels) or the first ten AP (bottom panels) were analyzed. PV-*Lmo4*KO showed increased width of AP (**b**), decreased decay of AP (**c**) and reduced fast afterhyperpolarization (fAHP). n=11 cells/5 WT, 13 cells/5 KO mice. ***, p<0.0001. (**e**) No difference in the I_A currents. 19 cells/4 WT, 12 cells/3 KO, 11 cells/3 DKO (PV-Cre/*Lmo4*$^{flox/flox}$*PTP1B*$^{flox/flox}$) mice. All data are presented as means ± SEM.

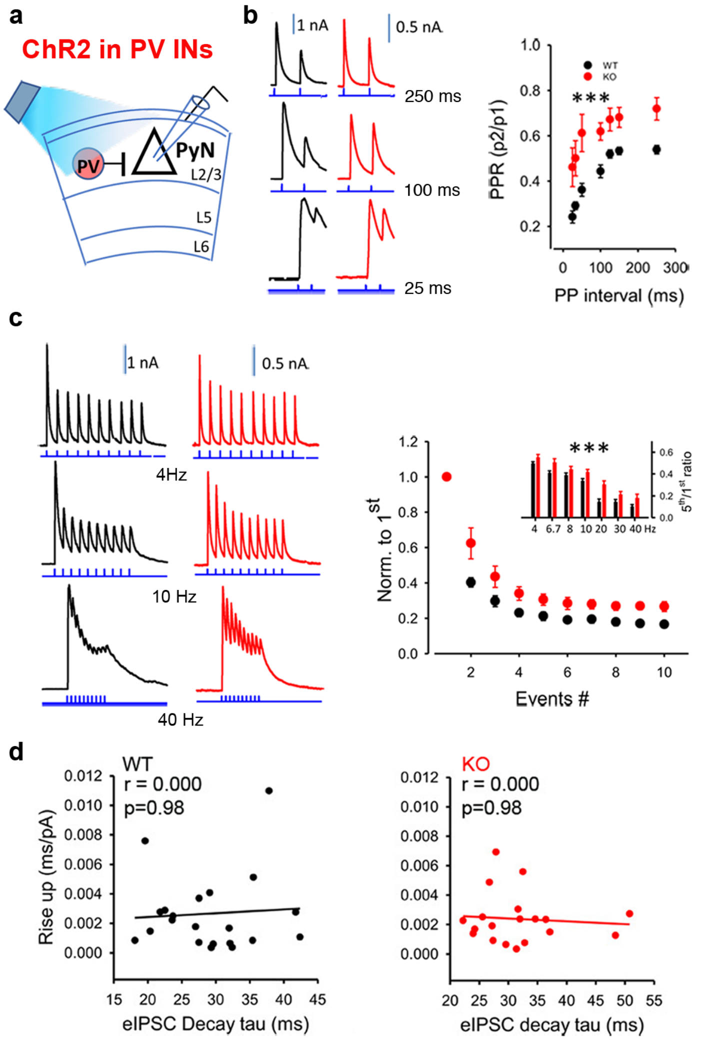

Supplementary Figure 4 Direct photo-activation of PV interneurons shows an increase in paired-pulse ratio (PPR) but decreased short-term depression of IPSC in PV-*Lmo4*KO mice. (**a**) AAV9 vectors expressing cre-dependent ChR2 were stereotactically injected to the dACC of PV-Cre/*Lmo4*KO$^{WT/WT}$ (WT) or PV-Cre/*Lmo4*$^{flox/flox}$ (PV-*Lmo4*KO) mice. The inhibitory synaptic responses at the L2/3 pyramidal neurons (PyN) after photoactivation of PV interneurons (PV INs) showed an increase in (**b**) PPR. The sample traces of WT (black) and PV-*Lmo4*KO (red) at various pulse durations were shown at the left. (**c**) Short-term depression of IPSC was also reduced in KO, as shown at 20 Hz. Left are sample traces at different frequencies. Inset, the mean of 5th/1st ratio of inhibitory synaptic responses were compared at various stimulation frequencies. n= 11 cells from 6 WT, 14 cells from 5 KO mice for **b** & **c**. (**d**) No correlation of IPSC decay time and rise slope further confirms these inhibitory inputs occur at the somata of L2/3 pyramidal neurons, consistent with where PV interneurons synapse. n = 22 cells/7 WT, 19 cells/7 KO mice. ***, p< 0.0001.

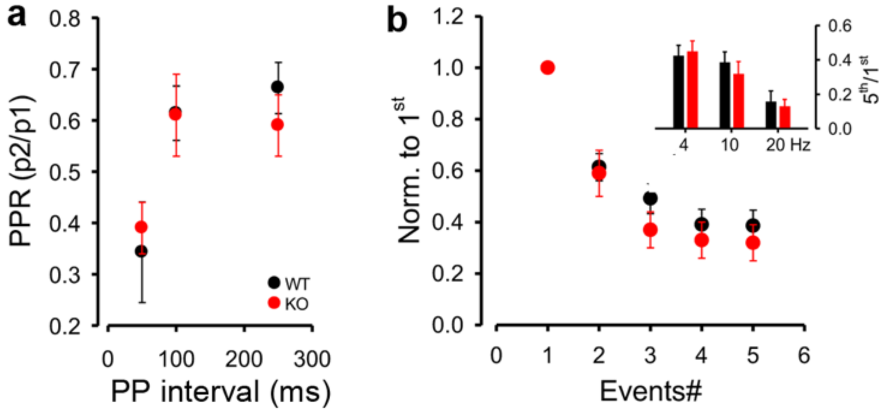

Supplementary Figure 5 No change in excitatory synaptic response at the dACC layer 2/3 pyramidal neurons of PV-*Lmo4*KO mice after photo-activation of MD thalamocortical projections. (**a**) Paired-pulse ratio of EPSC at various pulse durations. (**b**) Short term depression of EPSC after repetitive stimulation at 10 Hz, inset is the mean of the ratio of $5^{th}/1^{st}$ EPSC at 4, 10, 20 Hz. n= 13 cells/5 WT, 20 cells/6 KO mice.

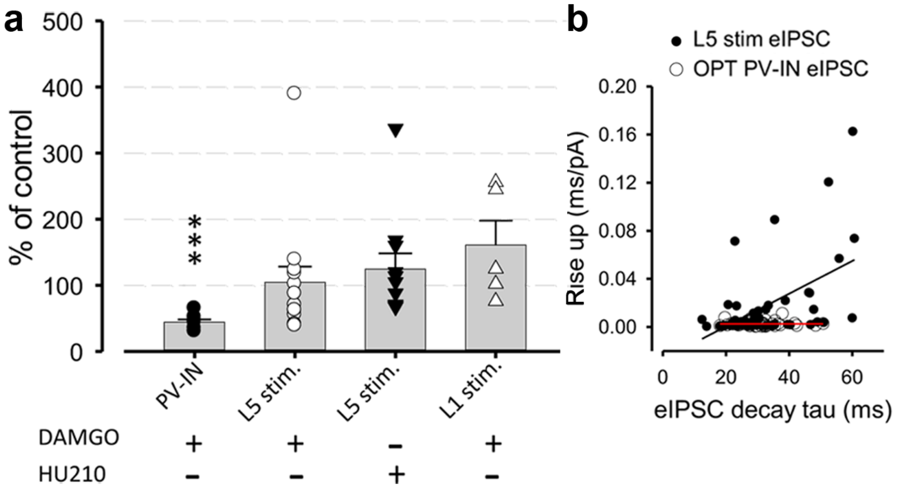

Supplementary Figure 6 The FFI IPSC employed by L1 and L5 inputs is not sensitive to DAMGO which inhibits synaptic GABA release from the PV interneurons, nor is it sensitive to HU210 (a CB1R agonist known to block synaptic release from CCK interneurons). (**a**) For any given L2/3 pyramidal neuron, the IPSC was obtained under no stimulation (i.e., spontaneous IPSC, sIPSC) or after photoactivation of PV interneurons or electrical stimulation at L1 or L5 (evoked IPSC, eIPSC). The changes of IPSC amplitudes after treatment with DAMGO or HU210 were compared, and normalized to before treatment and expressed in %. Optogenetic-induced PV-mediated IPSC was reduced to 44 ± 4.2% of control after DAMGO treatment. In contrast, the eIPSC elicited by L5 electrical stimulation was not sensitive to DAMGO or HU210, indicating that these eIPSC are mainly derived neither from PV nor CCK interneurons, respectively; these L5 stimulation-induced eIPSC are likely derived from SST interneurons. Similarly, L1 stimulation-induced eIPSC was not sensitive to DAMGO and was derived from non-PV interneurons, likely SST interneurons. (**b**) Correlation of IPSC decay time and rise slope indicates these L5 stimulated eIPSC inputs occur along the (distal) dendrites of L2/3 pyramidal neurons, in contrast to PV-mediated IPSCs (OPT PV-IN eIPSC) that occur at the somata of L2/3 pyramidal neurons ($R = 0.53$, $p < 0.0001$, n=51 cells from 14 WT mice for L5 eIPSC; $R = 0.0037$, $p = 0.98$, n=41 cells from 7 WT mice for OPT PV-IN eIPSC, optogenetic activation of PV interneurons). ***, $p<0.001$.

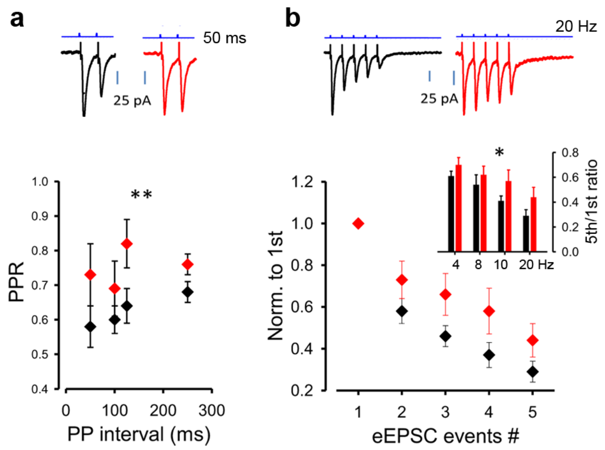

Supplementary Figure 7 The EPSC recorded in L2/3 pyramidal neurons in response to electrical stimulation at L5 showed an increase of PPR (**a**) and reduced short-term depression (**b**, 10 Hz) in PV-*Lmo4*KO mice. (**b**) Inset shows the mean of the ratio of $5^{th}/1^{st}$ IPSC or EPSC at various frequencies. Representative traces are shown above each graph (WT, black; KO red). n= 13 cells/5 WT, 19-24 cells/6 KO mice. *, **, p<0.05, 0.01, respectively.

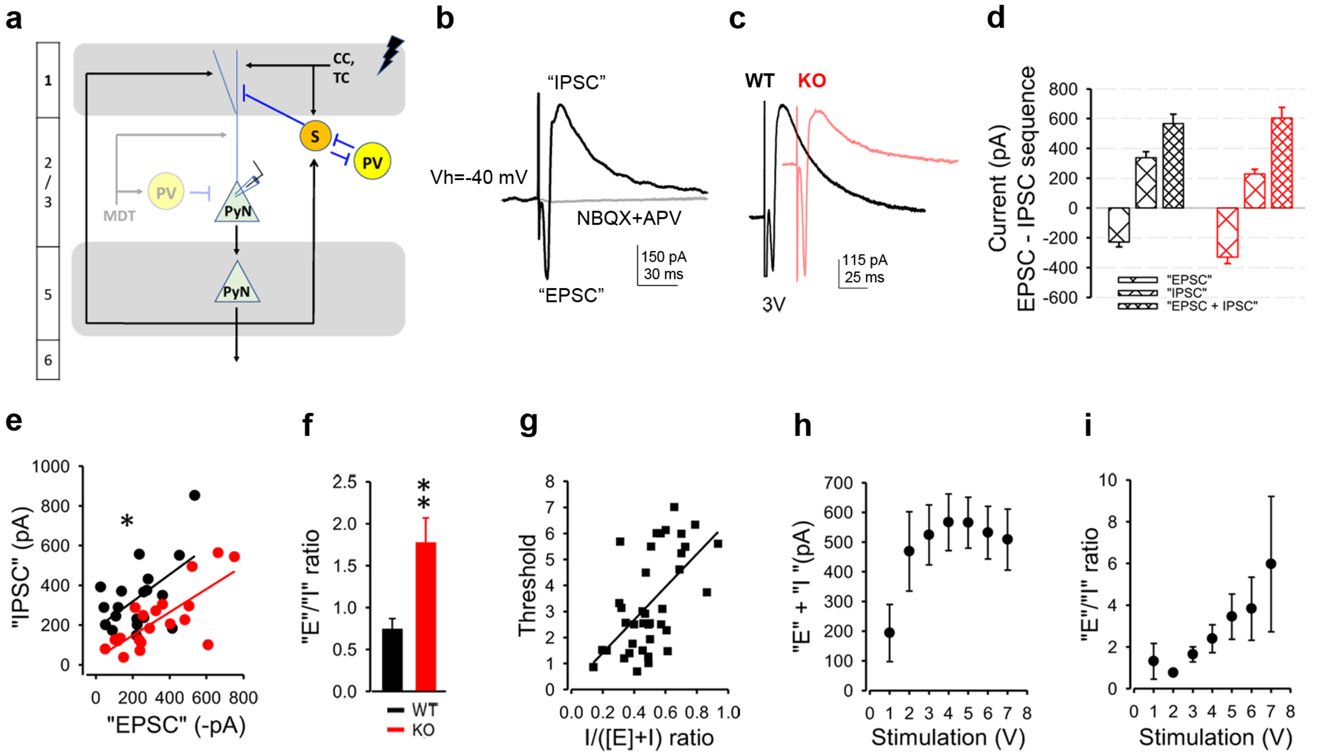

Supplementary Figure 8 Layer 1 electrical stimulation elicits a monosynaptic EPSC and a feedforward di-synaptic IPSC at the dACC layer 2/3 pyramidal neurons. The FFI was much increased in PV-*Lmo4*KO mice. (**a**) Diagram of the inhibitory circuits and placement of stimulation electrodes at layer 1 of the ACC. (**b, c**) The excitatory and inhibitory responses were recorded with voltage clamp at -40 mV as “EPSC”, “IPSC”, respectively. The representative traces were obtained after L1 electrical stimulation at ~3V. (**b**) Blocking the synaptic response with an AMPAR blocker NBQX and a NMDAR blocker APV abolished the FFI (in grey). (**c**) Example EPSC – IPSC sequence traces of WT (black) and KO (red) show that the sum of inhibitory and excitatory responses was not different between wild type and PV-*Lmo4*KO mice, but differed in the relative proportion of “IPSC” and “EPSC” amplitudes (**d**). (**e**) The amplitudes of “IPSC” relative to the “EPSC” fitted with liner regression showed a subtractive reduction in KO compared to WT (*, p=0.03 for intercept between WT and KO) and (**f**) the E/I ratio was much increased; this is similar to the results obtained with L5 electrical stimulation (see **Figure 5**). n=19 cells/8 WT, 22 cells/8 KO mice for **d**, **e**. **, 0.0023. (**g**) The threshold to elicit 50% of action potentials increases as a function of the ratio of inhibitory currents to total currents. (**h-i**) Under improved clamp preparation (with Cs^+ in the pipette solution), we further tested the EPSC – IPSC sequence with step increases in stimulation. Four WT L2/3 pyramidal cells were used to assess the stimulation threshold and the responses. The total (“EPSC”+”IPSC”) currents reached a plateau when stimulation reached ~3V (**h**), but the ratio of “EPSC”/”IPSC” increased with stimulation strength (**i**). n= 38 cells/14 WT mice for **g-i**.

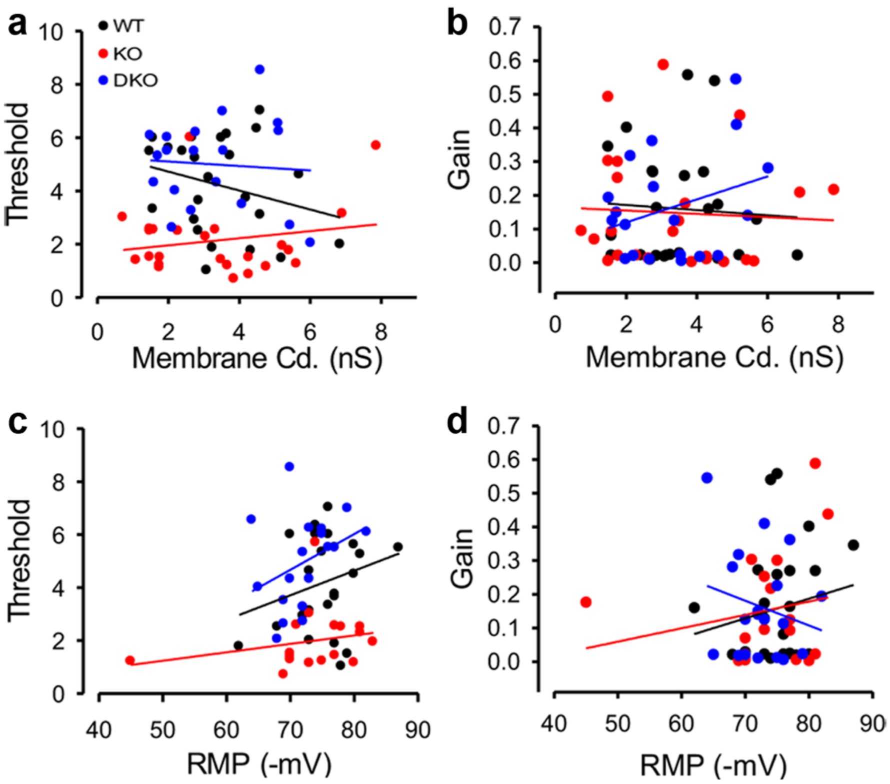

Supplementary Figure 9 The intrinsic properties of L2/3 pyramidal neurons recruited across ranges of input strengths and gains in response to L1 stimulation. The thresholds to recruit pyramidal neurons and their gains (output responses relative to input stimuli) showed no correlation with resting membrane conductance (**a, b**) or resting membrane potential (**c, d**). KO, PV-*Lmo4*KO. n= 24 cells/8 WT, 24 cells/8 KO, 19 cells/7 DKO mice for **a**-**d**.

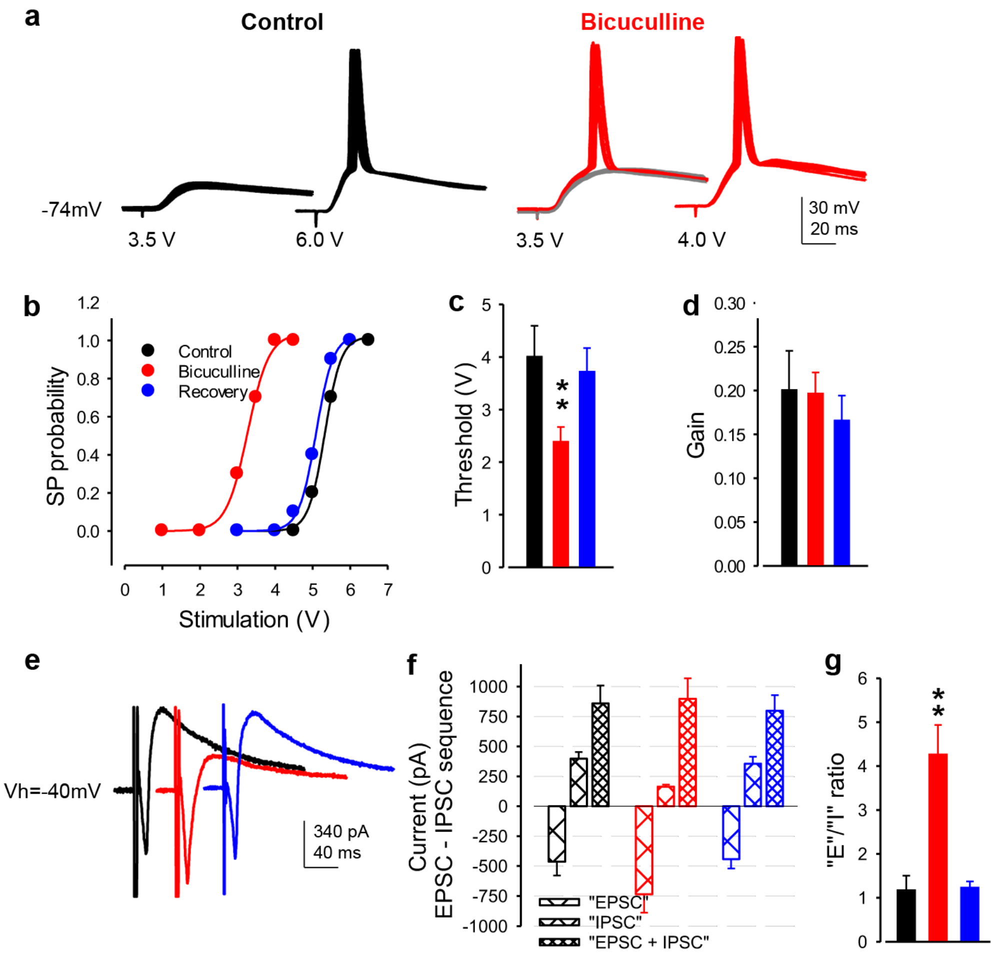

Supplementary Figure 10 Reduced threshold of layer 2/3 dACC pyramidal neurons in response to electrical stimulation at layer 1 observed in PV-*Lmo4*KO mice can be replicated by subthreshold inhibition of GABA receptors with 2 μM bicuculline (IC50) in wild type mice. (**a**) Sample traces of current clamp recording show that the stimulation threshold to elicit an action potential is markedly reduced from 6 V to 3.5 V in the present of bicuculline (2 μM); 10 sweeps are overlaid for each condition. (**b**) Representative data from an individual L2/3 pyramidal neuron before (black) and after bicuculline treatment (red) and washout (blue). The stimulation threshold to elicit AP (spike probability) is reduced (leftward shifted) in the presence of bicuculline and is reversed after washout. (**c**) Average thresholds to elicit AP. n=6 cells from 4 WT mice. (**d**) Gain was not affected by treatments. For each cell subjected for current clamp studied in **a-d**, we also performed a subsequent voltage clamp recording (**e-g**). (**e**) Sample traces under voltage clamp at -40 mV show the EPSC and di-synaptic feedforward IPSC recorded at a L2/3 pyramidal neuron before (black) and after bicuculline treatment (red) and washout (blue). (**f**) Despite the sum of inhibitory and excitatory responses was not different before and after bicuculline treatment, bicuculline treatment suppressed inhibitory inputs with increased EPSC and thereby elevated the “E”/”I” ratio (**g**). *, **, ***, p<0.05, 0.01, 0.005, respectively.

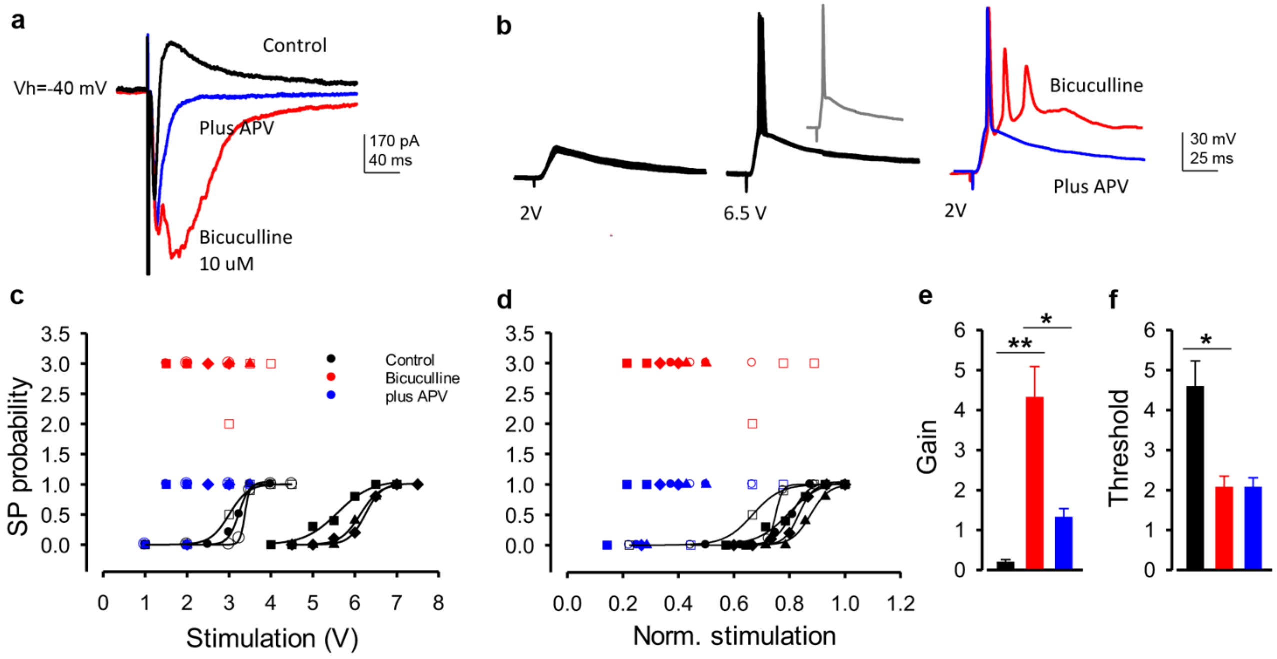

Supplementary Figure 11 High concentration (10 μM) of bicuculline completely blocks layer 1 GABAergic inputs and increases the spike probability. (**a**) Sample traces under voltage clamp at -40 mV show the monosynaptic EPSC and di-synaptic feedforward IPSC recorded at a L2/3 pyramidal neuron before (black) and after treatment with bicuculline (red) or bicuculline plus APV, an NMDAR blocker (blue). Complete blockade of GABAergic inputs produces larger EPSCs consisting an early AMPAR- and a late NMDAR-mediated EPSCs; the latter can be blocked by APV. (**b**) Sample traces of current clamp recording show that the stimulation threshold to elicit an action potential is further reduced from 6 V to 2 V in the present of bicuculline (10 μM). Moreover, bicuculline treatment led to bursting action potentials (red trace) that were abolished by APV (blue trace). (**c, d**) The AP probabilities over various stimulation strengths obtained from 6 cells before and after bicuculline and plus APV application. Only the control could be fitted to sigmoid function, while a direct linear fit was used to obtain the slope and thresholds for bicuculline-treated cells. Completely blockade of GABAergic inputs abolished the pyramidal cell's ability to fine tune its dynamic response, resulting in all-or-none AP bursting[10, 11]. Norm. stimulation, normalized stimulation. (**e**) 10 μM bicuculline markedly increases the gain and a majority of gain is mediated via NMDAR as it was diminished by addition of APV (blue). (**f**) Blocking GABAergic inputs lowered the thresholds of stimuli to elicit AP that were not affected by additional treatment with APV. *, **, $p<0.05$, 0.01. n=6 cells/4 WT/treatment for **c**-**f**.

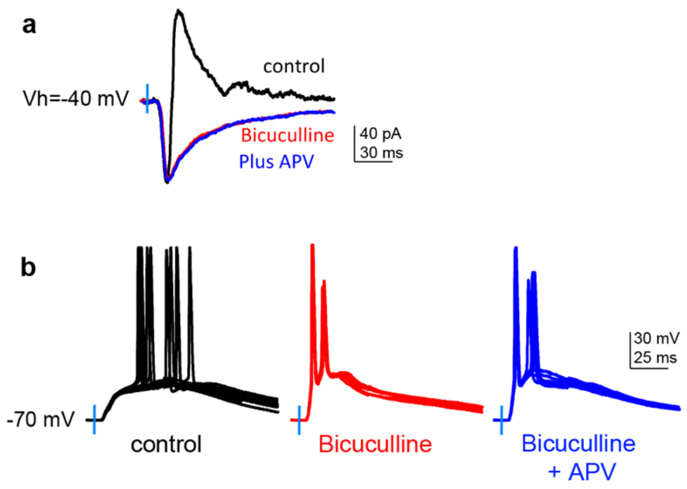

Supplementary Figure 12 High concentration (10 µM) of bicuculline completely blocked MD thalamocortical GABAergic inputs and increased L2/3 pyramidal neuron spike probability. (**a**) Sample traces under voltage clamp at -40 mV show the monosynaptic EPSC and di-synaptic feedforward IPSC recorded at a L2/3 pyramidal neuron before (black) and after treatment with bicuculline (red) or bicuculline plus an NMDAR blocker APV (blue). In contrast to L1 electrical stimulation (**Supplementary Fig. 11a**), blockade of MD thalamic GABAergic inputs revealed that the EPSC consisted of only an APV-insensitive AMPAR-mediated EPSC. (**b**) 10 sample traces of current clamp recording after each photostimulation before and after treatment with bicuculline (red) or bicuculline plus APV (blue) were overlaid and compared. Blocking GABAergic input increases the numbers of APs elicited (from 9 to 20 AP for total 10 recording sweeps) and APV treatment did not affect AP production, in contrast to what we observed during L1 electrical stimulation (**Supplementary Fig. 11b**). N=5 cells from 3 mice.

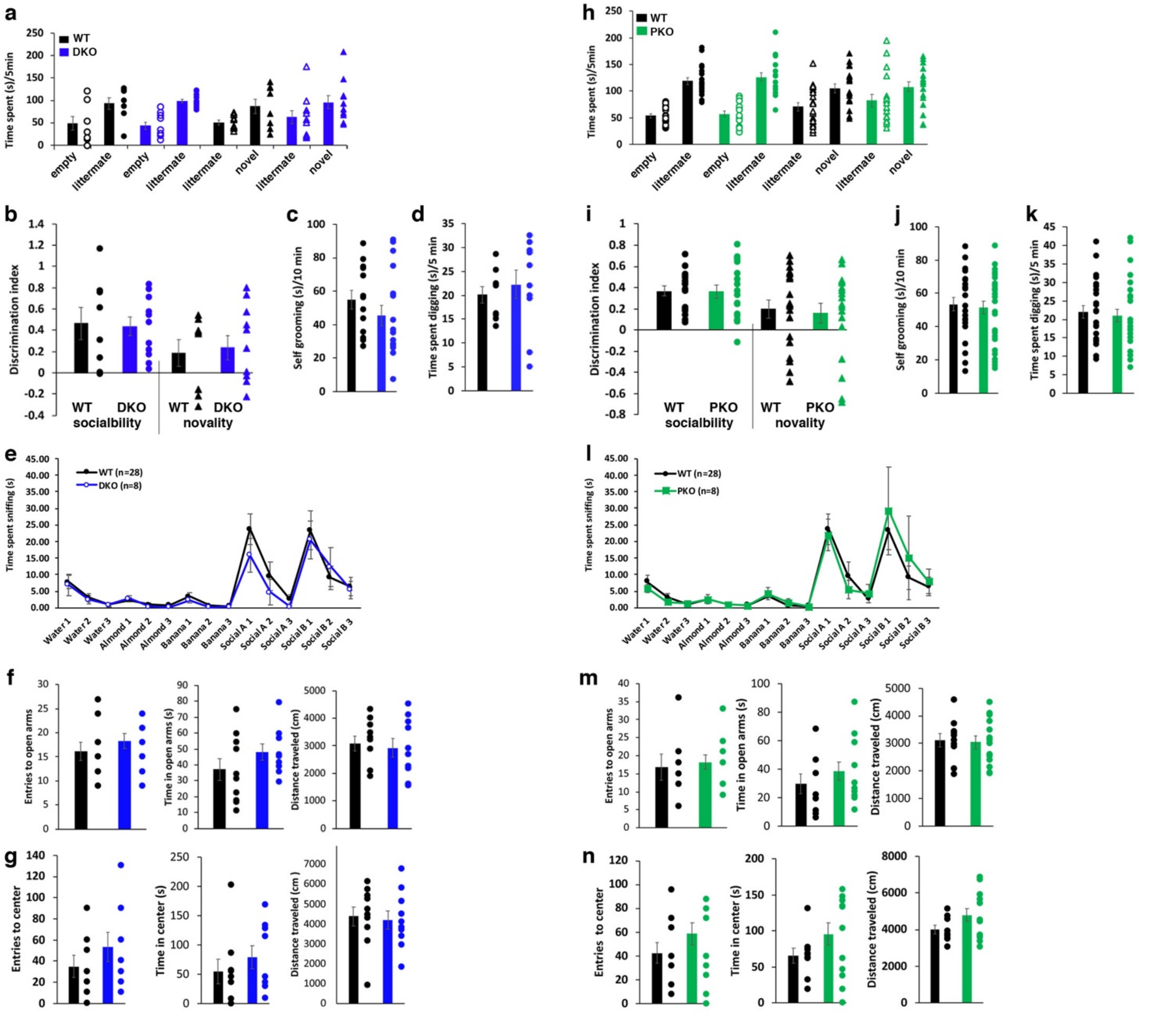

Supplementary Figure 13 Behavior tests of PV-DKO (**a-g**) and PV-PTP1BKO (PKO) mice (**h-n**). (**a**) Time spent in social interaction and novelty tests, used for discrimination index in (**b**) and Figure 7a. n=14 WT, 11 DKO. Time spent in (**c**) self-grooming and (**d**) digging. (**e**) Olfactory response measured as time spent sniffing non-social and social odors. n=28 WT, 8 DKO. (**f**) Elevated plus maze test. (**g**) Open field test. n=10 WT, 9 DKO (d, e). (**h-n**) Same behavior tests carried out for PKO mice. n= 19 WT, 18 PKO (h, i), 24 WT, 29 PKO (j, k), 28 WT, 9 PKO (l), 9WT, 13 PKO (m, n).

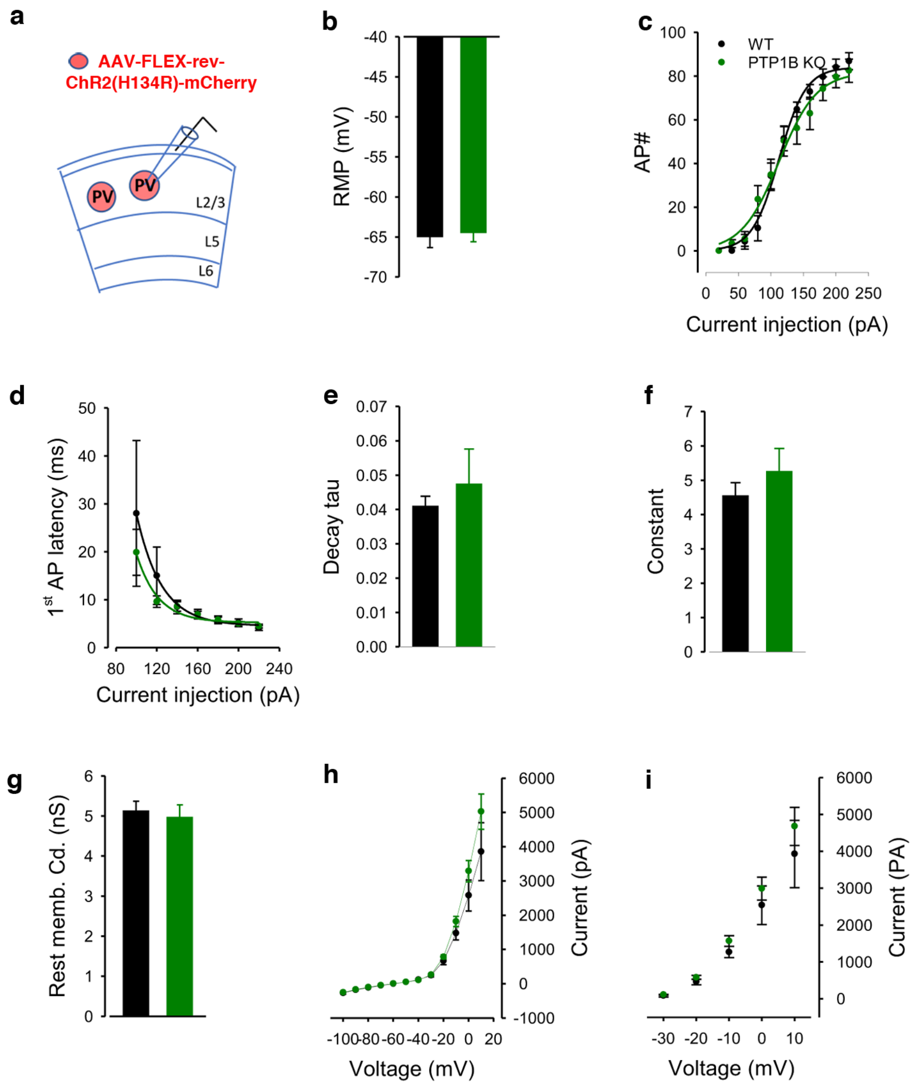

Supplementary Figure 14 PV interneuron properties are normal in PV-PTP1BKO mice. (**a**) Diagram of AAV9 vectors expressing cre-dependent mCherry stereotactically injected to the dACC of WT (PV-Cre/PTP1B$^{WT/WT}$) or PKO (PV-Cre/PTP1B$^{flox/flox}$) mice to label PV neurons. (**b**) Resting membrane potential, (**c**) membrane excitability (current injection–action potential curve, fitted to a sigmoidal function. (**d-f**) Latency to first action potential. Current injection–1st AP latency, fitted to a three-parameter exponential decay, revealed a similar decay tau (**e**), and time constant (**f**). (**g**) Resting membrane conductance. (**h, i**) I-V curves: WT (black) and PKO (green); **i**, isolated delayed rectified potassium currents. n= 11 cells/4 WT, 32 cells/5 PKO mice for **b-i**.

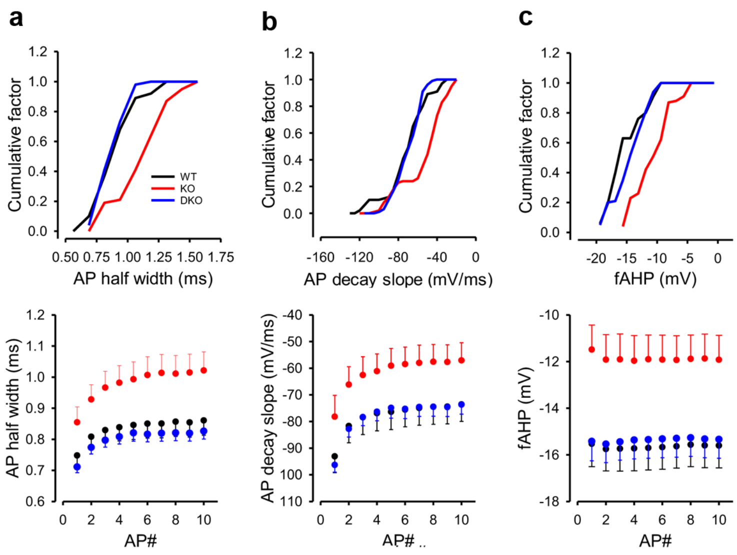

Supplementary Figure 15 Most PV interneuron properties are normalized in PV-DKO mice, including action potential width (**a**), decay slope (**b**), and the fast afterhyperpolarization (fAHP) (**c**). n= 11 cells/5 WT, 13 cells/5 KO (PV-*Lmo4*KO), 20 cells/7 DKO mice.

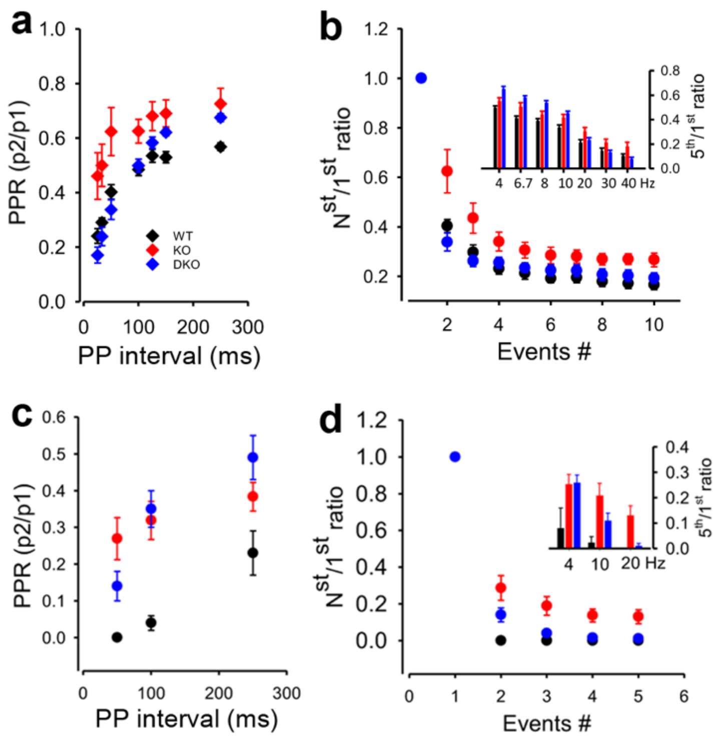

Supplementary Figure 16 PV-DKO mice normalized PV-mediated inhibitory inputs onto layer 2/3 pyramidal neurons either by direct photo-activation of PV interneurons (**a**, **b**) or by indirect photo-activation of thalamocortical projections to the dACC (**c**, **d**). (**a**, **c**) The paired pulse ratio and (**b, d**) short-term depression of inhibitory currents are shown. n= 11 cells/4 WT, 14 cells/ 5 KO, 18 cells/6 DKO mice.

Supplementary Table 1 Results of statistical analysis for main figures.

Fig.		t or z	p	n	Anova
1a	Sociability: 0.44 ± 0.05, 0.20 ± 0.06	2.647	0.0155	8 WT, 14 KO	
	Novelty: 0.21 ± 0.08; -0.8 ± 0.09	2.240	0.0379	7 WT, 13 KO	
1b	Grooming: 57.71 ± 6.71, 115.4 ± 12.93	-3.483	0.0033	7 WT, 10 KO	
1c	Digging: 14.88 ± 3.40, 31.36 ± 5.34	-2.376	0.0290	8 WT, 11 KO	
1d	cFos+ neurons in ACC			4WT NSI, 4WT SI, 4KO NSI, 4KO SI	(see below in Fig 7c)
2b	RMP: WT: -65.4 ± 1.3, KO: -58.8 ± 2.1 mV	-2.650	0.0150		
2c	threshold: WT, 112.3±2.4; KO, 91.1±2.4 pA	-6.130	< 0.0001		
	increased slope: WT, 20.6±2.0; KO, 26.1±2.1,	1.900	0.0300	9-11 cells/5 WT, 18-3 cells/5 KO mice	
	max. AP#: WT, 83.9±2.1; KO, 91.2±1.9,	2.510	0.0060		
2e	WT, 0.0412±0.0027; KO, 0.0202±0.0042 ms/pA	4.200	< 0.0001		
2f	time constant: WT, 4.56±0.36; KO, 3.11±0.47 ms	-2.450	0.0070		
2g	Cd (nS) : WT 5.1 ± 0.2 ; KO 3.8 ± 0.4	3.000	0.0075		
2 i					Genotypes F(1,110) = 6.22, p = 0.015
					Genotypes*Voltages F(4,110) = 1.45, p = 0.226
3c inset	WT 52.53 ± 4.12 , KO 90.1 ± 11.3; MW U 205		< 0.001		
3d inset	WT 23.96 ± 1.08, KO 23.47 ± 1.90; MW U 348		0.2660		
3e inset	WT 5.96 ± 0.43, KO 4.93 ± 0.49; MW U 322.5		0.1310		
3f inset	WT 31.1 ± 3.2 , KO 26.1 ± 1.86; MW U 352.5		0.2970		
3g inset	WT 8.08 ± 0.56, KO 7.9 ± 0.46; MW U 413.5		0.9260		
3h inset	WT 6.14 ± 1.31, KO 6.39 ± 1.26; MW U 406		0.8340		
3i	linear regression (R = 0.64, p < 0.001 for WT; R = 0.61, p < 0.001 for KO) were observed in KO			26 cells/8 WT, 24 cells/8 KO mice	
	slope: WT: 0.88 ± 0.20, KO: 3.46 ± 0.88	2.860	0.0021		
	intercept: WT 22.9 ± 6.7, KO: -2.8 ± 23.4	-1.100	0.1500		
3j	sEPSC WT -31.1 ± 3.2, KO -26.1 ± 1.86 pA	-1.300	0.1970		
	sIPSC WT 52.5 ± 4.1, KO 90.1 ± 11.7 pA	3.220	0.0023		
3k	E/I ratio WT 0.62 ± 0.05, KO 0.34 ± 0.03	1.690	0.0048		
4c	E-I relationsip R = 0.76, p < 0.001 for WT; R = 0.9, p < 0.0001 for KO			21 cells/ 7 WT, 23 cells/ 8 KO	
	The slope, WT:3.0 ± 0.6 , KO: 14.5 ± 1.8	2.550	0.0054		
	The intercept, WT: 76.4 ± 71.0, KO: 38.7 ± 169.0	-0.090	0.4600		
4d	eEPSC WT 78.8 ± 21.4, KO 80.0 ± 15.2	-0.050	0.9600		
	eIPSC WT: 282.5 ± 82.2 pA, KO: 1129.3 ± 247.1 pA	-3.100	0.0035		
4e	E/I ratio WT 0.49 ± 0.11, KO 0.11 ± 0.01	3.690	0.0006		
4f	Latency WT: 3.51 ± 0.43 , KO: 2.17 ± 0.39	2.300	0.0265		
4h	PPR		0.0000		Genotypes*pulse _duration F(2,129) = 1.23, p = 0.3; Genotype F(1,129) = 35.2, p < 0.00001
4j	short-term depression		0.0007		Genotype*frequency F(1,129) = 0.47, p = 0.63, Genotype F(1,129) = 12.1, p = 0.0007 (5th/1st ratio)
			< 0.0001		4, 10 and 20 Hz, F(1,26) = 240, 121 and 113, p < 0.0001 for all (repeat measurement)
4n	maximum spike probability WT: 1.24 ± 0.11, KO: 0.80 ± 0.08, MW U = 22		0.0150	15 cells/6WT, 8 cells/4KO mice	
4o	stimulation threshold WT: 535.6 ± 41.2, KO: 237.8 ± 30.0 mA.ms	-5.85	< 0.0001		
4p	gain WT: 0.0024 ± 0.0003, KO: 0.0101 ± 0.0031, MW U = 17.5		0.0070		
5c	slope: WT: 2.05 ± 0.69, KO: 1.68 ± 0.58	-0.400	0.3400	38 cel/s/117 WT, 13 cells/5 KO mice	
	intercept: WT: 1115.1 ± 234.8, KO: -44.3 ± 203.9	-3.7	0.0001		
5d	excitatory inputs WT: 255 ± 37, KO: 297 ± 54 pA, MW U=190.5		0.1900		
	inhibitory inputs WT: 1638 ± 170.8, KO: 457.3 ± 137 pA, MW U=57		<0.001		
5e	E/I ratio WT: 0.185 ± 0.019; KO: 0.895 ± 0.124, MW U = 17		<0.001		
5f	latency WT 2.89 ± 0.30, KO 2.90 ± 0.35 ms	-0.00875	0.99		
5g, h	PPR			14 cells/5 WT, 11 cells/5 KO mice	genotype*pulse_duration F(3,92) = 0.18, p =0.90; genotype F(1,92) = 7.94, p = 0.0018.
	short term plasticity				genotype*frequency F(3,92) = 0.14, p =0.94; genotype F(1,92) = 6.2, p = 0.015).
6e	threshold, WT: 4.2 ± 0.4, KO: 2.1 ± 0.3	4.5	< 0.0001	24 cell/8WT, 24 cells/8KO mice	
6f	gain, WT: 0.16 ± 0.035, KO: 0.15 ± 0.035, MW U = 247		0.4		

7a	Sociability	-0.361	0.7230	14 WT, 14 LKO, 11 DKO	F(2, 36)=3.919, p=0.0288; Tukey post-hoc WT:0.483 ± 0.076; LKO: 0.201 ± 0.059; DKO: 0.424 ± 0.086; WT vs LKO: p=0.034; WT vs DKO: p>0.999
	Novelty	-0.381	0.7080	15 WT, 13 LKO, 11 DKO	F(2, 36)=3.917, p=0.029; Tukey post-hoc WT:0.198 ± 0.075; LKO: -0.085 ± 0.086; DKO: 0.239 ± 0.113; WT vs LKO: p=0.0297; WT vs DKO: p=0.051
7b	Grooming	1.089	0.2860	20 WT, 10 LKO, 17 DKO	F(2, 44)=22.697, p=1.68705E-07; Tukey post-hoc WT:55.931 ± 4.298; LKO: -115.4 ± 12.927; DKO: 45.538 ± 6.182; WT vs LKO: p<0.0001; WT vs DKO: p=0.4093
	Digging	-0.590	0.5630	17 WT, 11 LKO, 10 DKO	F(2, 35)=4.473, p=0.0186; post-hoc WT:17.656 ± 1.899; LKO: -31.363 ± 5.343; DKO: 22.230 ± 2.996; WT vs LKO: p=0.0098; WT vs DKO: p=0.5299
7c	c-Fos+ neurons in ACC. WT NSI: 1 ± 0.49, WT SI: 8.11 ± 1.48; KO NSI: 0.01 ± 0.01; KO SI 3.71 ± 0.23; DKO NSI 0 ± 0; DKO SI 5.04 ± 0.61			4 WT NSI, 4WT SI, 4LKO NSI, 4LKO SI, 4DKO NSI, 4DKO SI	SI: F (1, 18) = 87.52, P < 0.0001; genotype: F (2, 18) = 8.263,P = 0.0028; interaction:F (2, 18) = 3.100,P = 0.0697. Post-hoc Bonferroni's multiple comparisons test:NSI:WT vs. SI:WT, p< 0.0001; NSI:LKO vs. SI:LKO, p=0.0206; DKO NSI vs DKO SI, p=0.0010; WT SI vs LKO SI, p=0.0041. WT ST vs DKO SI, p=0.0840.
7d	RMP			9-11 cells/ 5 WT, 8-13 cells/5 KO, 16-20 cells/7 DKO	F (2, 41) = 5.3, p = 0.009; post-hoc WT: -65 ± 1.3, DKO: -65 ± 1.2 mV, t = 0.16, p = 1.0
7e	threshold: WT, 112.3 ± 2.5; DKO, 126.3 ± 3.1 pA	-3.500	0.0001		
	maximum AP#: WT, 84 ± 2.1; DKO, 83 ± 2.6	-0.290	0.3900		
7f	decay tau WT, 0.04 ± 0.003; DKO, 0.033 ± 0.010	-0.800	0.2200		
	constant: WT, 4.6 ± 0.4; DKO, 5.6 ± 1.0	0.990	0.1600		
7g	Rest membrane conductance				F (2, 41) = 4.4, p = 0.019; post-hoc WT: 5.14 ± 0.23, DKO: 4.93 ± 0.35 nS, t = 0.44, p = 1.0
7i	isolated delayed rectifiers				Genotypes F(1,145) = 19.92, p = 1.74 x 10*-5; Genotypes*Voltages F(4,145) = 4.04, p = 0.00398
8a	E-I relationsip R = 0.76, p < 0.001 for WT; R = 0.76, p < 0.0001 for DKO			21 cells/ 7 WT, 23 cell/8 KO, 35 cells/12 DKO mice	
	The slope, WT: 3.0 ± 0.6, DKO: 2.30 ± 0.334	-1.015	0.4600		
	The intercept, WT: 76.4 ± 71.0, DKO: 69.4 ± 106	-0.055	0.4800		
8b	E/I ratio				F (2, 70)= 6.3, p=0.003, post-hoc WT: 0.49 ± 0.11, DKO: 0.56 ± 0.10, t=0.47, p = 1.0
8c	latency				F (2, 70)=4.0, p=0.024, post-hoc WT: 3.5 ± 0.4, DKO: 3.3 ± 0.3 ms, t = 0.43, p = 1.0
8f	Max SP proability			15cells/5WT, 8cells	H(2)=10.44, p=0.005; WT: 1.24 ± 0.11, DKO: 1.11 ± 0.06, Q=0.94, p = 1.0; KO:0.80 ± 0.08, DKO: 1.11 ± 0.06, Q=3.10, p=0.006
8g	threshold				F(2,29)=19.034, p < 0.001; WT: 237.8 ± 30.0, DKO: 231.6 ± 42.1 mA.mS, t = 0.12, p = 1.0; KO: 535.6 ± 41.2, DKO:231.6 ± 42.1 mA.ms, t=5.25, p<0.001
8h	gain				H(2)=9.64, p=0.008, WT: $1.0x10^{-2}$, DKO: $7.7x10^{-3}$, Q=0.29, p=1.0; KO: $2.4x10^{-3}$, DKO:$7.7x10^{-3}$, t=2.75, p=0.018.
8k	gain (L1 input)			24cells/8WT,24cell	H(2)=0.83, p=0.66
8l	threshold (L1 input)				F(2,64)=18.6 , p<0.001; WT: 4.2 ± 0.4, DKO: 5.0 ± 0.4 V, t=1.6, p=0.34; KO:2.1 ± 0.3, DKO: 5.0 ± 0.4, t=5.8, p<0.001

Supplementary Table 2 Results of statistical analysis for supplementary figures.

Fig.		t or z	p	n	dF	
S1	OF (Entries): 50.82 ± 2.68; 50.67 ± 3.68	0.035	0.9720	17 WT, 12 LKO	27	ttest
S1	OF (Time): 86.42 ± 7.59; 87.93 ± 11.70	-0.113	0.9106	16 WT, 12 LKO	26	ttest
S1	OF (Distance): 4399 ± 153; 4591 ± 235	-0.71687	0.479394	18 WT, 12 LKO	28	ttest
S1	EPM (Entries): 15.00 ± 1.49; 12.67 ± 1.78	1.00225	0.324804	18 WT, 12 LKO	28	ttest
S1	EPM (Time): 42.25 ± 8.32; 44.77 ± 7.48	-0.2115	0.834029	18 WT, 12 LKO	28	ttest
S1	EPM (Distance): 3117 ± 89; 2939 ± 123	1.19556	0.242264	18 WT, 11 LKO	27	ttest
S1	Olfactory function (see S13)			28 WT, 12 LKO		
S3b, c, d	ANOVA: (**b**) genotype*AP# F(9,190) = 0.07, p = 0.99; genotype F(2,190) = 42.4, p < 0.0001)). (**c**) genotype*AP# F(9,190) = 0.007, p = 0.99; genotype F(1, 190) = 30.7, p < 0.0001. (**d**) genotype*AP# F(9, 190) = 0.006, p = 0.99; genotype F(1, 190) = 71.1, p < 0.00001)			11 cells/5 WT, 10 cells/5 KO mice		
S3e	ANOVA Genotypes F(2,351) = 0.29, p = 0.745; Genotypes*Voltages F(16,351) = 0.08, p = 0.999			19 cells/4 WT, 12cells/3 KO, 11cells/3 DKO mice		
S4	(**b**) PPR: ANOVA, genotype*pulse_duration F(6,200) = 0.33, p = 0.92; genotype F(1,200) = 56.6, p < 0.0001.			19cells/8WT,16 cells/8KO mice for PPR. 11 cells/6 WT, 14 cells/5 KO mice for STD		
	(**c**) Short-term depression: ANOVA, genotype*frequency F(6,174) = 0.22, p = 0.97; genotype F(1,174) = 28.9, p < 0.0001.					
	(**c**) repeated ANOVA for all frequencies F(1,23) = 4,3,7.3, 4.7, 6.9 ,6.0, 5.5, 4.8, p < 0.05 for all.					
	(d) IPSC decay time vs. rise slope			22 cells/7 WT, 19 cells/7 KO mice		
S5	(**a**) genotype*pulse_duration F(2,81) = 0.36, p = 0.7, genotype F(1,81) = 0.016, p = 0.90			15 cells/5 WT, 11cells/6 KO mice		
	(**b**) genotype**frequency F(2,71) = 0.29, p = 0.75, genotype F(1,71) = 0.2, p = 0.650					
S6	(**a**) DMAGO: OPT PV-IN 44 ± 4.2%	13.200	< 0.0001	8 cells/4 WT mice		
	(**a**) DMAGO: L5 stimulation: 104.4 ± 23.6%	-0.19	0.85	14 cells/5 WT mice		
	(**a**) HU210: L5 stimulation: 124.5 ± 28.8%	-1.030	0.3300	11 cells/5 WT mice		
	(**a**) DAMGO L1 stimulation: 161.1 ± 37.0%	-1.600	0.1800	6 cells/4 WT mice		
	(**b**) Correlation of IPSC decay time vs. rise slope: R = 0.53, p < 0.0001, for L5 eIPSC			51 cells/14 WT mice		
	(**b**) Correlation of IPSC decay time vs. rise slope: R = 0.0037, p = 0.98, for OPT PV-IN eIPSC (optogenetic activation of PV interneurons)			41 cells/7 WT mice		
S7	(**a**) ANOVA, genotype*pulse_duration F(3,93) = 0.31, p =0.81; genotype F(1,93) = 9.4, p = 0.0029. (**b**) ANOVA, genotype*frequency F(3,93) = 0.43, p =0.73; genotype F(1,93) = 5.2, p = 0.0025.			15 cells/5WT, 11 cells/6KO mice		
S8	(**e**) The amplitudes of "IPSC" relative to the "EPSC" fitted with liner regression: R = 0.56 m, p = 0.012 for WT; R = 0.75, p = 0.0001 for KO. (slope: WT: 0.68 ± 0.24, KO: 0.58 ± 0.12, Z = -0.35, p = 0.36; intercept: WT: 183.0 ± 64.8, KO: 31.1 ± 46.7, Z = -1.9, p = 0.03)			19 cells/8 WT, 22 cells/8 KO mice		
	(g) threshold/(I([E]+I)): R = 0.59, p < 0.0001					
	(f) "E/I" ratio WT 0.75 ± 0.12, KO 1.82 ± 0.30	-3.270	0.0023			
S9	(**a**) threshold: Cd: WT R = 0.26, p = 0.21; KO R = 0.18, p = 0.36; DKO R = 0.07, p = 0.77.			24 cells/8 WT,24 cells/8 KO, 19 cells/7 DKO mice		
	(**b**)gain: WT R = 0.06, p = 0.78; KO R = 0.06, p = 0.80; DKO R = 0.31, p = 0.20)					
	(**c**) threshold: RMP: WT R = 0.25, p = 0.24; KO R = 0.23, p = 0.35; DKO R = 0.36, p = 0.13.					
	(d) gain: RMP: WT R = 0.17, p = 0.44; KO R = 0.19, p = 0.44; DKO R = 0.21, p = 0.38).					
S10	(**c**) AP thresholds WT 4.01 ± 0.58, bicucu 2.40 ± 0.27, recovery 3.73 ± 0.43	3.830	0.0044	6 cells/4 WT mice		
	(**d**) gain WT 0.20 ± 0.04, bicucu 0.20 ± 0.023, recovery 0.17 ± 0.028	0.092	0.9300			
	(**g**) "E/I" ratio WT 1.19 ± 0.023, bicucu 4.28 ± 0.66, recovery 1.25 ± 0.13	-6.100	0.0017			
S11	(**e**) gain control 0.206 ± 0. 0.047, bicucu 4.33 ± 0.76, plus APV 1.33 ± 0.2			6 cells/4 WT mice/treatment		
	control to bicu	-5.520	0.0027			
	recovery to bicucu	-2.880	0.0340			
	(f) threshould control 4.60 ± 0. 0.63, bicucu 2.08 ± 0.27, plus APV 2.08 ± 0.23					
	control to bicuc	3.568	0.0160			
	recovery to bicucu	0.000	1.0000			

	Sociability: WT: 0.369 ± 0.043, PKO 0.365 ± 0.062	-0.06344	0.949774	19 WT, 18 PKO	35	ttest
	Novety: WT:0.199 ± 0.086, 0.159 ± 0.097	0.3672	0.71568	19 WT, 18 PKO	35	ttest
	Grroming: WT=53.446 ± 4.012 PKO=51.413 ± 3.914	0.358	0.7220	24 WT, 29 PKO	51	ttest
	Digging: WT=22.1 ± 1.714, PKO=21.055 ± 1.771	0.423	6743	24 WT, 29 PKO	51	ttest
S13	OF (Entries): 35.00 ± 10.52; 53.33 ± 14.04	-1.02309	0.3225	8 WT, 9 DKO	15	ttest
	OF (Time): 54.81 ± 20.90; 79.19 ± 19.14	-0.860	0.4020	9 WT, 9 DKO	16	ttest
	OF (Distance): 4392 ± 476; 4207 ± 450	0.281	0.7814	10 WT, 10 DKO	18	ttest
	EPM (Entries): 16.2 ± 1.91; 18.3 ± 24.9	-0.848	0.4070	10 WT, 10 DKO	18	ttest
	EPM (Time): 37.08 ± 6.77; 48.03 ± 5.05	-1.272	0.2200	10 WT, 9 DKO	17	ttest
	EPM (Distance): 3080 ± 277; 2920 ± 333	0.369	0.7164	10 WT, 10 DKO	18	ttest
	Olfactor function (see below)			28 WT, 8 DKO		
S13	OF (Entries): 42.4 ± 8.52; 58.46 ± 9.07	-1.257	0.2220	10 WT, 13 PKO	21	ttest
	OF (Time): 65.99 ± 10.42; 95.63 ± 15.46	-1.439	0.1654	9 WT, 13 PKO	20	ttest
	OF (Distance): 4000 ± 226; 4787 ± 354	-1.683	0.1078	9 WT, 13 PKO	20	ttest
	EPM (Entries): 16.8 ± 3.58; 18.23 ± 1.99	-0.370	0.7150	10 WT, 13 PKO	21	ttest
	EPM (Time): 29.36 ± 7.06; 38.55 ± 6.41	-0.947	0.3550	9 WT, 13 PKO	20	ttest
	EPM (Distance): 3114 ± 246; 3038 ± 225	0.226	0.8230	10 WT, 14 PKO	22	ttest
	Olfactor function (see below)			28 WT, 8 PKO		
S1 & 13	Olfactory functions: genotyes* odors F (42, 728) = 0.4083, P = 0.9997; odor tirals: F (14, 728) = 15.24, P < 0.0001; genotypes: F (3, 52) = 0.2097, P = 0.8892			28 WT, 12 LKO, 8 DKO, 8 PKO		
S14	b) RMP -65.0 ± 1.3 (WT) to -64.5 ± 1.1 mV,t = -0.28, p = 0.78			9-11 cells/5 WT, 22 -32 cells/ 8 PTP1B KO mice		
	c) threshould (50% of maximum) 112.3 ± 2.4 (WT) to 112.4 ± 4.9	0.022	0.49			
	maximum spike # 84 ± 2.1 (WT) to 81.7 ± 3.8 (PTP1B KO)	-0.53	0.3			
	Slope 20.6 ± 2.0 (WT) to 28.9 ± 3.8 (PTP1B KO)	1.91	0.028			
	e) decay tau 0.0411 ± 00028 (WT) to 0.0475 ± 0.0101 (PTP1B KO)	-0.61	0.37			
	f) constant 4.56 ± 0.37 (WT) to 5.27 ± 0.66 (PTP1B KO)	0.94	0.17			
	g) Rest membrane conductance (nS)					
	5.14 ± 0.23 (WT) to 4.98 ± 0.30 (PTP1B KO), t = 0.46, p = 0.65	0.46	0.65			
	i) ANOVA Genotypes F(1,195) = 1.60, p = 0.207; Genotypes*Voltages F(4,195) = 0.24, 0.915					
S15	(a) AP width (ANOVA genotypes*AP# F (18, 340) = 0.069, p = 1.0; genotypes F(2, 340) = 55.5, p < 0.0001; post-hoc DKO to WT p = 0.15, DKO to KO p < 0.0001),			11 cells/5 WT, 10 cells/5 KO, 16 cells/7 DKO mice.		
	(b) decay slope (ANOVA genotypes*AP# F (18, 340) = 0.018, p = 1.0; genotypes F(2, 340) = 29.4, p < 0.0001; post-hoc DKO to WT p = 0.94, DKO to KO p < 0.0001),					
	(c) fast afterhyperpolarization (fAHP)(ANOVA genotypes*AP# F (18, 340) = 0.01, p = 1.0; genotypes F(2, 340) = 46.3, p < 0.0001; post-hoc DKO to WT p = 1, DKO to KO p < 0.0001).					
S16						
S16 a						
PPR	genotypes F (2,319) = 44.4, p < 0.0001			19 cells/5 WT, 16cells/4 KO, 18 cells/5 DKO mice		
	Genotypes*Frequency F(6,319) = 2.07, p = 0.018					
	post-hoc WT to DKO p = 1; DKO to KO p < 0.0001.					
S16 b						
5th/1st ratio	genotypes F (2,279) = 25.6, p < 0.0001			11 cells/5 WT, 14cells/4 KO, 18 cells/5 DKO mice		
	Genotypes*Frequency F(6,279) = 5.6, p < 0.0001					
	post-hoc WT to DKO p < 0.0001; DKO to KO p = 1.					
PPR				21 cells/7 WT, 21cells/8 KO, 19 cell/8 DKO mice		
S16 c	Genotypes F(2,165) = 20.87, p = 8.33 x 10*-9					
	Genotypes* duration F(4,165) = 2.26, p = 0.065					
	post-hocwt to DKO p = 7.22 x 10*-7, DKO to KO p = 1.					
5th/1st ratio	Genotypes F(2,127) = 5.97, p = 0.00332			6 cells/3 WT, 21 cells/8 KO, 19 cell/8 DKO mice		
S16 d	Genotypes* frequency F(4,127) = 1.00, p = 0.407					

Supplementary References:

1. Hippenmeyer, S., *et al.* A developmental switch in the response of DRG neurons to ETS transcription factor signaling. *PLoS Biol* **3**, e159 (2005).
2. Hahm, K., *et al.* Defective neural tube closure and anteroposterior patterning in mice lacking the LIM protein LMO4 or its interacting partner Deaf-1. *Mol Cell Biol* **24**, 2074-2082 (2004).
3. Bence, K.K., *et al.* Neuronal PTP1B regulates body weight, adiposity and leptin action. *Nat Med* **12**, 917-924 (2006).
4. Yang, M. & Crawley, J.N. Simple behavioral assessment of mouse olfaction. *Curr Protoc Neurosci* **Chapter 8**, Unit 8 24 (2009).
5. Zaman, T., *et al.* LMO4 Is Essential for Paraventricular Hypothalamic Neuronal Activity and Calcium Channel Expression to Prevent Hyperphagia. *J Neurosci* **34**, 140-148 (2014).
6. Qin, Z., *et al.* Chronic Stress Induces Anxiety via an Amygdalar Intracellular Cascade that Impairs Endocannabinoid Signaling. *Neuron* **85**, 1319-1331 (2015).
7. Kuroda, M., Yokofujita, J. & Murakami, K. An ultrastructural study of the neural circuit between the prefrontal cortex and the mediodorsal nucleus of the thalamus. *Prog Neurobiol* **54**, 417-458 (1998).
8. Collins, D.P., Anastasiades, P.G., Marlin, J.J. & Carter, A.G. Reciprocal Circuits Linking the Prefrontal Cortex with Dorsal and Ventral Thalamic Nuclei. *Neuron* **98**, 366-379 e364 (2018).
9. Pouille, F., *et al.* Input normalization by global feedforward inhibition expands cortical dynamic range. *Nat Neurosci* **12**, 1577-1585 (2009).
10. Kanter, E.D., Kapur, A. & Haberly, L.B. A dendritic GABAA-mediated IPSP regulates facilitation of NMDA-mediated responses to burst stimulation of afferent fibers in piriform cortex. *J Neurosci* **16**, 307-312 (1996).
11. Royer, S., *et al.* Control of timing, rate and bursts of hippocampal place cells by dendritic and somatic inhibition. *Nat Neurosci* **15**, 769-775 (2012).
