# Supplementary material for: Hyperactivated PTP1B phosphatase in parvalbumin neurons alters anterior cingulate inhibitory circuits and induces autism-like behaviors
Source: Nat Commun. 2020 Feb 24;11:1017. doi: 10.1038/s41467-020-14813-z (PMC7039907; doi:10.1038/s41467-020-14813-z)
Supplement: Supplementary file 3 — Reporting Summary [file 41467_2020_14813_MOESM3_ESM.pdf]

nature research

Corresponding author(s): Hsiao-Huei Chen
Li Zhang
Alexandre FR Stewart

Last updated by author(s): Dec 18, 2019

Reporting Summary

Nature Research wishes to improve the reproducibility of the work that we publish. This form provides structure for consistency and transparency in reporting. For further information on Nature Research policies, see Authors & Referees and the Editorial Policy Checklist .

Statistics

For all statistical analyses, confirm that the following items are present in the figure legend, table legend, main text, or Methods section.

n/a	Confirmed	
☐	☒	The exact sample size (*n*) for each experimental group/condition, given as a discrete number and unit of measurement
☐	☒	A statement on whether measurements were taken from distinct samples or whether the same sample was measured repeatedly
☐	☒	The statistical test(s) used AND whether they are one- or two-sided<br>*Only common tests should be described solely by name; describe more complex techniques in the Methods section.*
☐	☒	A description of all covariates tested
☐	☒	A description of any assumptions or corrections, such as tests of normality and adjustment for multiple comparisons
☐	☒	A full description of the statistical parameters including central tendency (e.g. means) or other basic estimates (e.g. regression coefficient) AND variation (e.g. standard deviation) or associated estimates of uncertainty (e.g. confidence intervals)
☐	☒	For null hypothesis testing, the test statistic (e.g. *F*, *t*, *r*) with confidence intervals, effect sizes, degrees of freedom and *P* value noted<br>*Give P values as exact values whenever suitable.*
☒	☐	For Bayesian analysis, information on the choice of priors and Markov chain Monte Carlo settings
☒	☐	For hierarchical and complex designs, identification of the appropriate level for tests and full reporting of outcomes
☒	☐	Estimates of effect sizes (e.g. Cohen's *d*, Pearson's *r*), indicating how they were calculated

Our web collection on statistics for biologists contains articles on many of the points above.

Software and code

Policy information about availability of computer code

Data collection	in vitro data were collected in Clampfit 10.4. Animal behaviors were collected using Ethovision video tracking software (Noldus).
Data analysis	in vitro data were analyzed in Clampfit 10.4. Statistic analyses were performed using Sigmaplot 14.

For manuscripts utilizing custom algorithms or software that are central to the research but not yet described in published literature, software must be made available to editors/reviewers. We strongly encourage code deposition in a community repository (e.g. GitHub). See the Nature Research guidelines for submitting code & software for further information.

Data

Policy information about availability of data

All manuscripts must include a data availability statement. This statement should provide the following information, where applicable:

- Accession codes, unique identifiers, or web links for publicly available datasets
- A list of figures that have associated raw data
- A description of any restrictions on data availability

The data that support the findings of this study are available from the corresponding authors upon reasonable request.

Field-specific reporting

Please select the one below that is the best fit for your research. If you are not sure, read the appropriate sections before making your selection.

☒ Life sciences ☐ Behavioural & social sciences ☐ Ecological, evolutionary & environmental sciences

For a reference copy of the document with all sections, see nature.com/documents/nr-reporting-summary-flat.pdf

Life sciences study design

All studies must disclose on these points even when the disclosure is negative.

Sample size	Sample size for each experiment is indicated in the figure legend for each experiment. The sample size was chosen based on previous experience for each experiment to yield high power to detect specific effects (Qin et al, JN 2012; Qin et al, Neuron, 2015). No statistical methods were used to predetermine sample size.
Data exclusions	no data were excluded except unhealthy cells for EP recording as described in methods: Series resistance was compensated at ~50% and monitored during the process, cells with series resistances larger than 25 MΩ were excluded.
Replication	All attempts for replication were successful. Numbers of cells and mice for replicates are indicated in the figure legends.
Randomization	Animals were chosen based on correct genotypes. Animal pairs from the same litters were compared to decrease variance in age and rearing. Each experiment contained animals from at least two different litters to ensure that the differences can be observed in different litters. Within each group, all mice and cells were randomly selected.
Blinding	Investigators are blinded for genotypes for electrophysiological recording, behvaior and immunohistological studies.

Reporting for specific materials, systems and methods

We require information from authors about some types of materials, experimental systems and methods used in many studies. Here, indicate whether each material, system or method listed is relevant to your study. If you are not sure if a list item applies to your research, read the appropriate section before selecting a response.

Materials & experimental systems

n/a	Involved in the study	
☐	☒	Antibodies
☒	☐	Eukaryotic cell lines
☒	☐	Palaeontology
☐	☒	Animals and other organisms
☒	☐	Human research participants
☒	☐	Clinical data

Methods

n/a	Involved in the study	
☒	☐	ChIP-seq
☒	☐	Flow cytometry
☒	☐	MRI-based neuroimaging

Antibodies

Antibodies used	Parvalbumin (Swant PV235, 500x), the neuronal marker NeuN (Millipore MAB377, anti-mouse, 500x), cFos (Santa Cruz, sc52, rabbit, 50x) Cy2-, Cy3-conjugated secondary antibodies (Jackson Labs, 1000x dilution).
Validation	Cruz et al, 2017 Frontiers Cell Neu. PMID: 28769762; Schwaller B., et al. 1999, Am. J. Physiol. PMID: 9950767

Animals and other organisms

Policy information about studies involving animals; ARRIVE guidelines recommended for reporting animal research

Laboratory animals	Experimental mice included both males and females, between the ages of 2-4 months. PV-Cre mice (gift from Dr. Sylvia Arber, Jackson Lab # 017320) to LMO4flox/flox mice (gift from Dr. Jane Visvader). PTP1Bflox/flox mice (gift from Dr. Benjamin Neel, Jackson Lab #012679). All mice were bred on the C57BL6 background for over 12 generations.
Wild animals	NA.
Field-collected samples	NA.
Ethics oversight	All procedures for animal use were approved by the University of Ottawa Animal Care and Veterinary Service, and were performed according to institutional guidelines and in accordance with those of the Canadian Council on Animal Care.

Note that full information on the approval of the study protocol must also be provided in the manuscript.
